# Supplementary material for: Genome-Wide Identification and Expression Profiles of 13 Key Structural Gene Families Involved in the Biosynthesis of Rice Flavonoid Scaffolds
Source: Genes (Basel). 2022 Feb 24;13(3):410. doi: 10.3390/genes13030410 (PMC8951560; doi:10.3390/genes13030410)
Supplement: Supplementary file 1 [file genes-13-00410-s001.zip › Supplementary Figures.pdf]

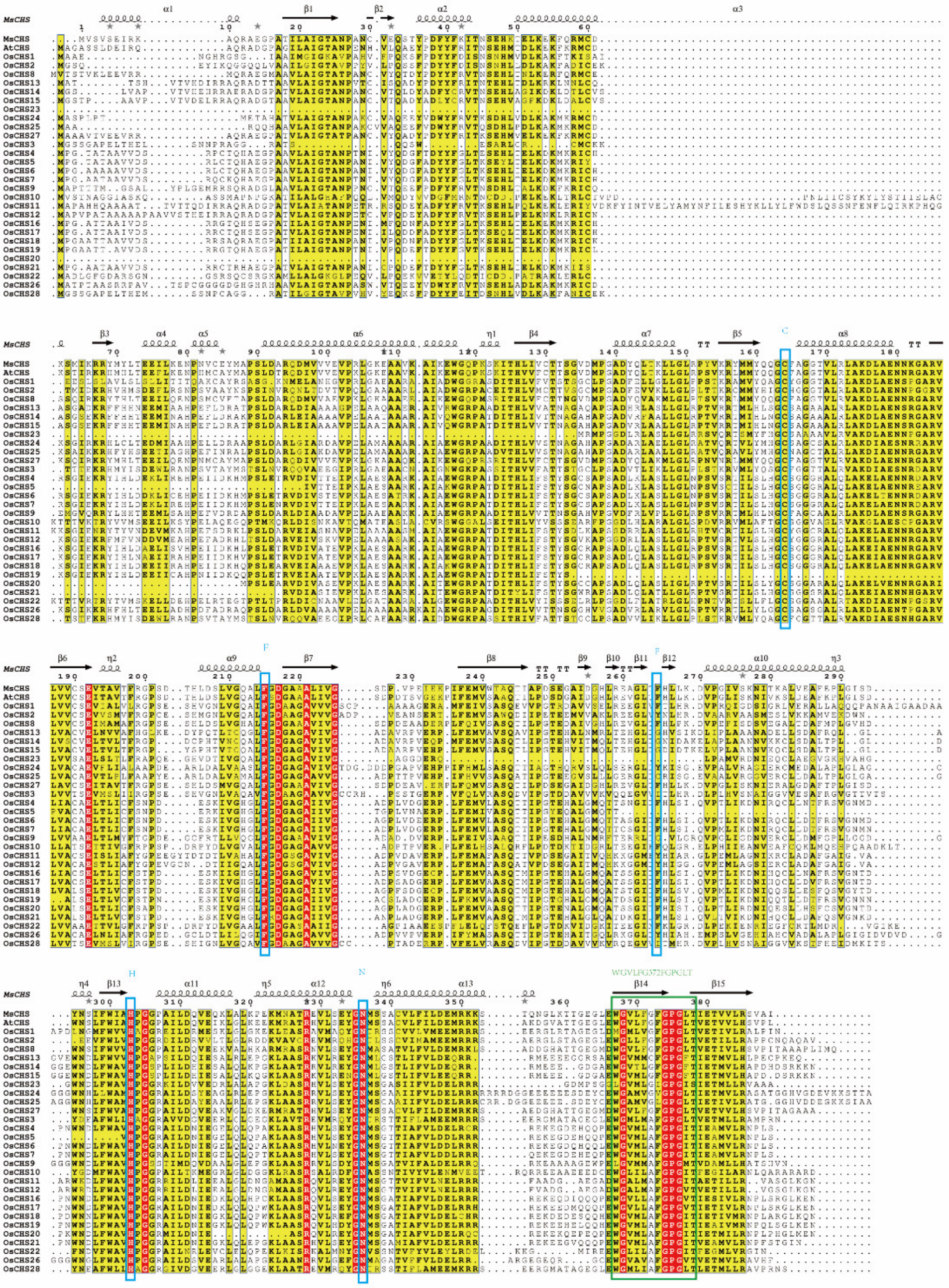

Supplementary Figure S1. Protein sequence alignment of OsCHSs against *Arabidopsis thaliana* CHS (AtCHS) *and Medicago sativa* CHS (MsCHS). The first line indicated the secondary structure of *MsCHS*. The red region represented strict sequence conservation regions. The yellow region represented relatively conserved regions. The blue boxes and letters indicated the catalytic triad (C164-H303-N336) and the gatekeeper (F) connected with CoA-binding. The green boxes and letters represented the highly conserved CHS characteristic signature sequence.

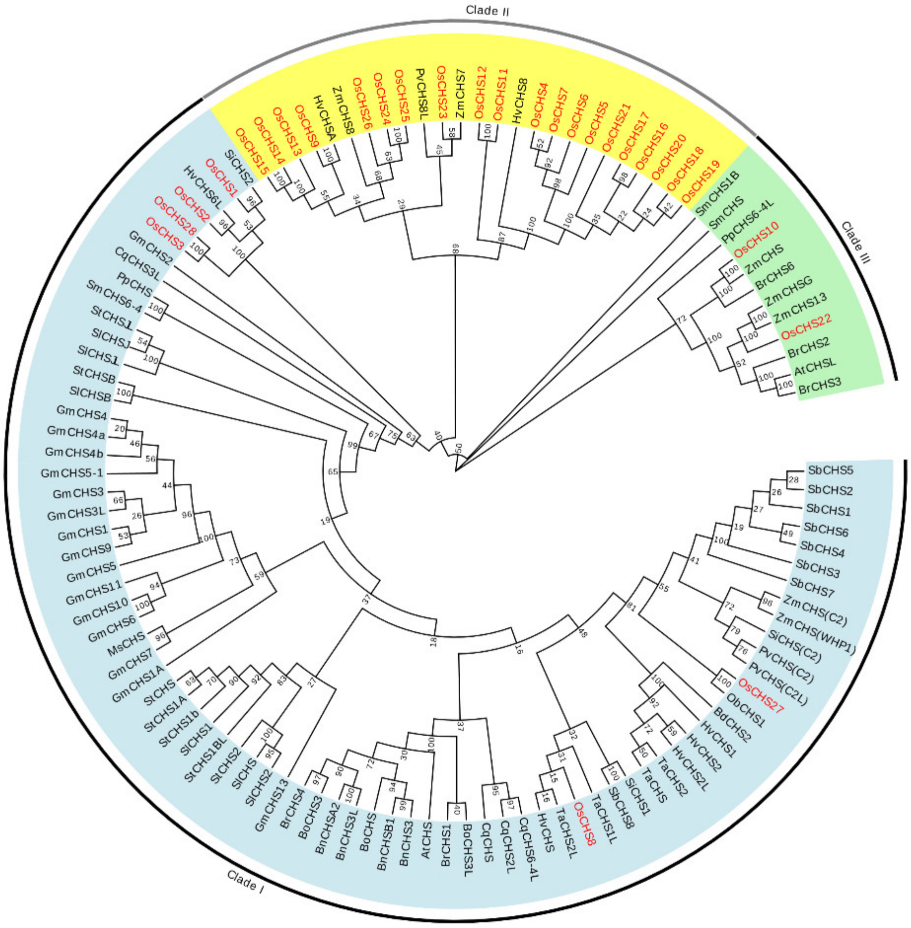

Supplementary Figure S2. Phylogenetic tree of the CHS proteins. The unrooted neighbor-joining tree was established by the MEGA7.0 program with 1000 Bootstrap replicates. The numbers at the nodes indicate the bootstrap values. Different colors of arcs represented distinct subfamilies. These CHS proteins used for phylogenetic tree were downloaded from NCBI and their accession numbers were list in Supplementary Table S1.

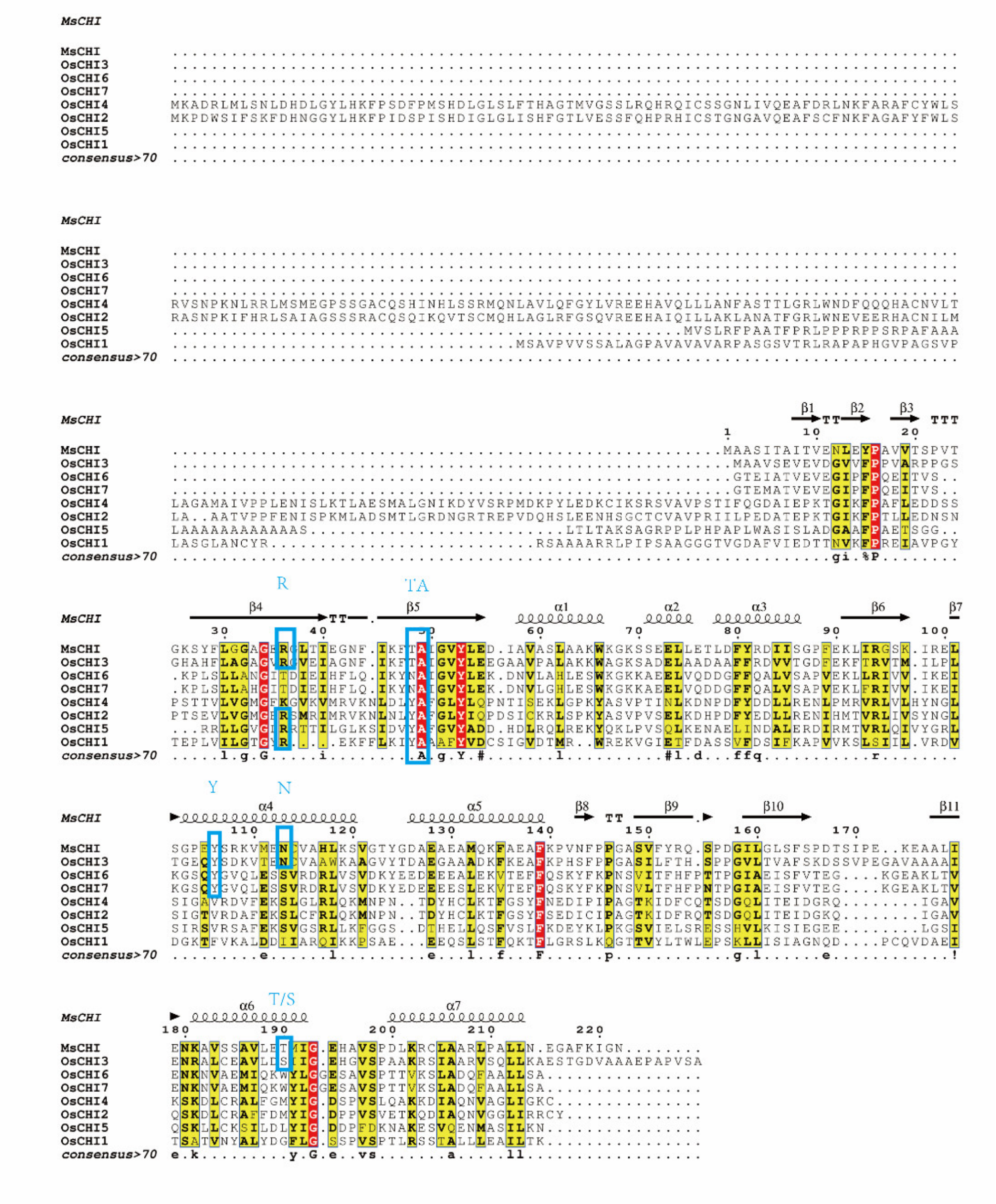

Supplementary Figure S3. Protein sequence alignment of OsCHIs against MsCHI. The first line indicated the secondary structure of MsCHI. The red region represented strict sequence conservation regions. The yellow region represented relatively conserved regions. The blue boxes and letters indicated the critical catalytic residues essential for CHI active sites R36, T48, Y106, N113. T190 is specific for legume, whereas S190 is more conserved among the non-legume plant species.

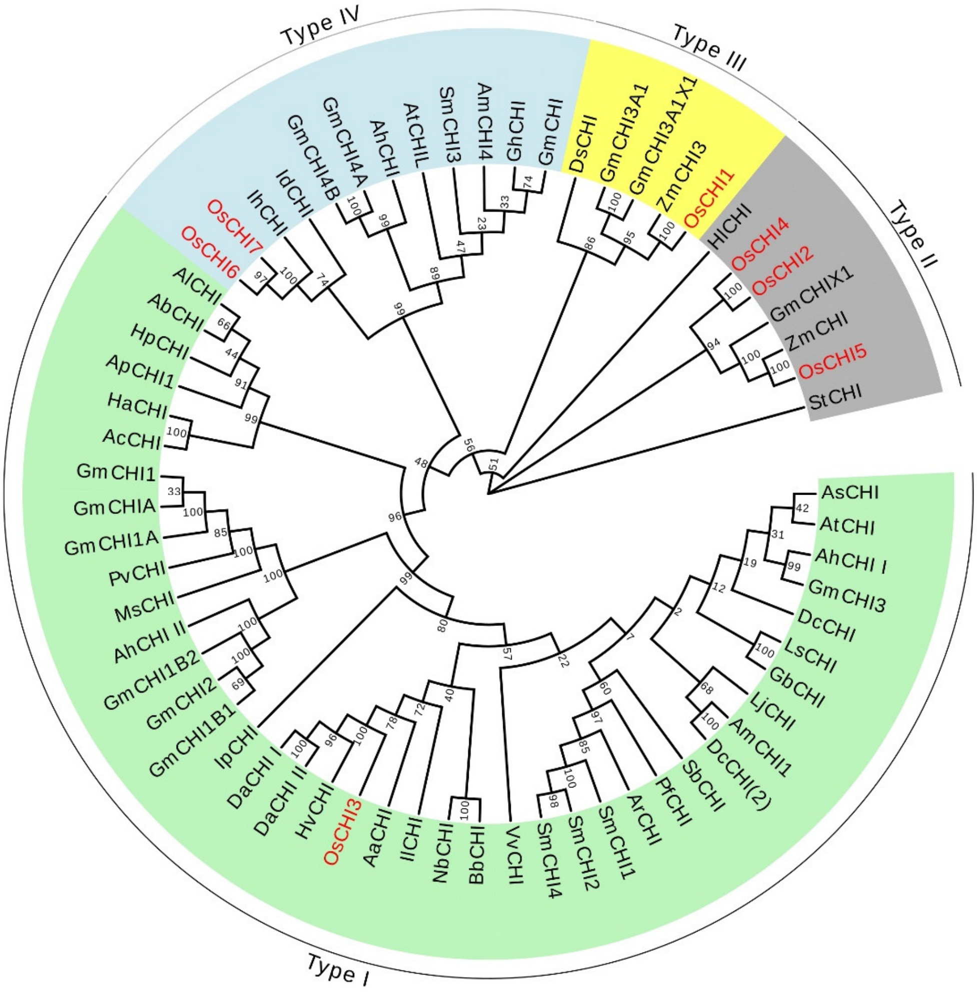

Supplementary Figure S4. Phylogenetic tree of the CHI proteins. The unrooted neighbor-joining tree was established by the MEGA7.0 program with 1000 Bootstrap replicates. The numbers at the nodes indicate the bootstrap values. Different colors of arcs represented distinct CHI types . These CHI proteins used for phylogenetic tree were downloaded from NCBI and their accession numbers were list in Supplementary Table S2.

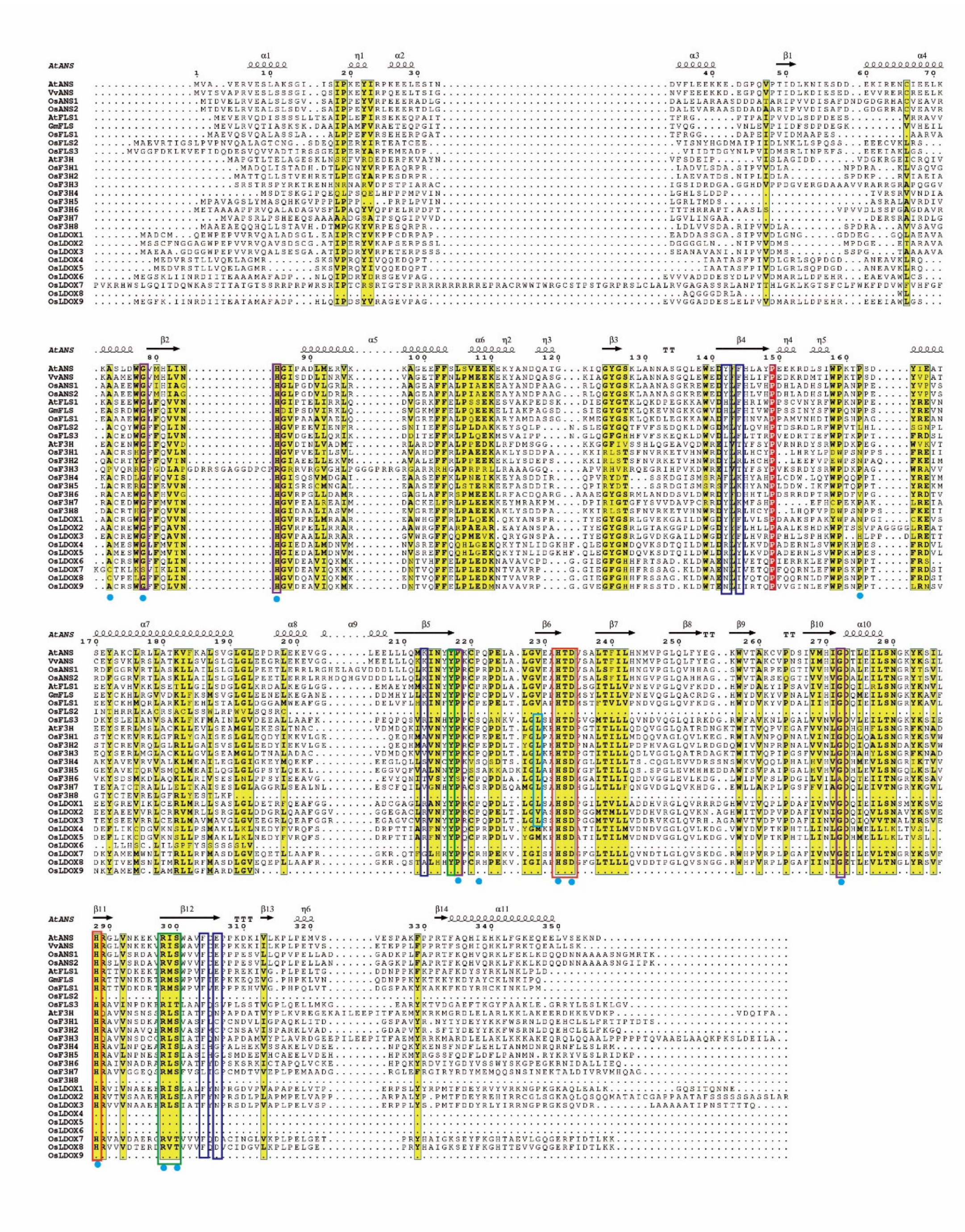

Supplementary Figure S5. Protein sequence alignment of flavonoid biosynthesis-related 2OGDs, including *A*NSs, FLSs, F3Hs and LDOXs. The first line indicated the secondary structure of AtANS. The red region represented strict sequence conservation regions. The yellow region represented relatively conserved regions. Residues responsible for binding ferrous iron, 2-oxoglutarate and substrate were remarked with red, green and dark-blue boxes, respectively. The residues important for proper folding of the 2OGD polypeptide were remarked with purple boxes. Residues relevant for F3H activity were marked with light-blue circles and boxes.

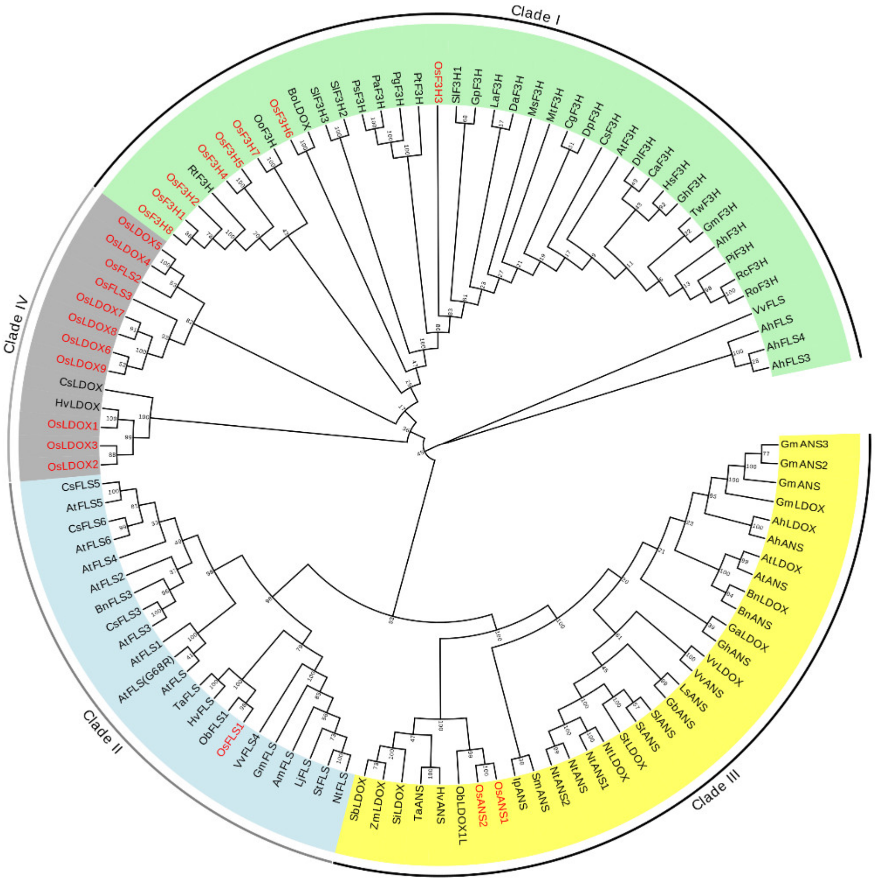

Supplementary Figure S6. Phylogenetic tree of the F3H, FLS, LDOX and ANS proteins. The unrooted neighbor-joining tree was established by the MEGA7.0 program with 1000 Bootstrap replicates. The numbers at the nodes indicate the bootstrap values. Different colors of arcs represented distinct clades. These flavonoid biosynthesis-related 2OGD proteins including ANSs, FLSs, F3Hs *and* LDOXs used for phylogenetic tree, were retrieved from NCBI and their accession numbers were list in Supplementary Table S3.

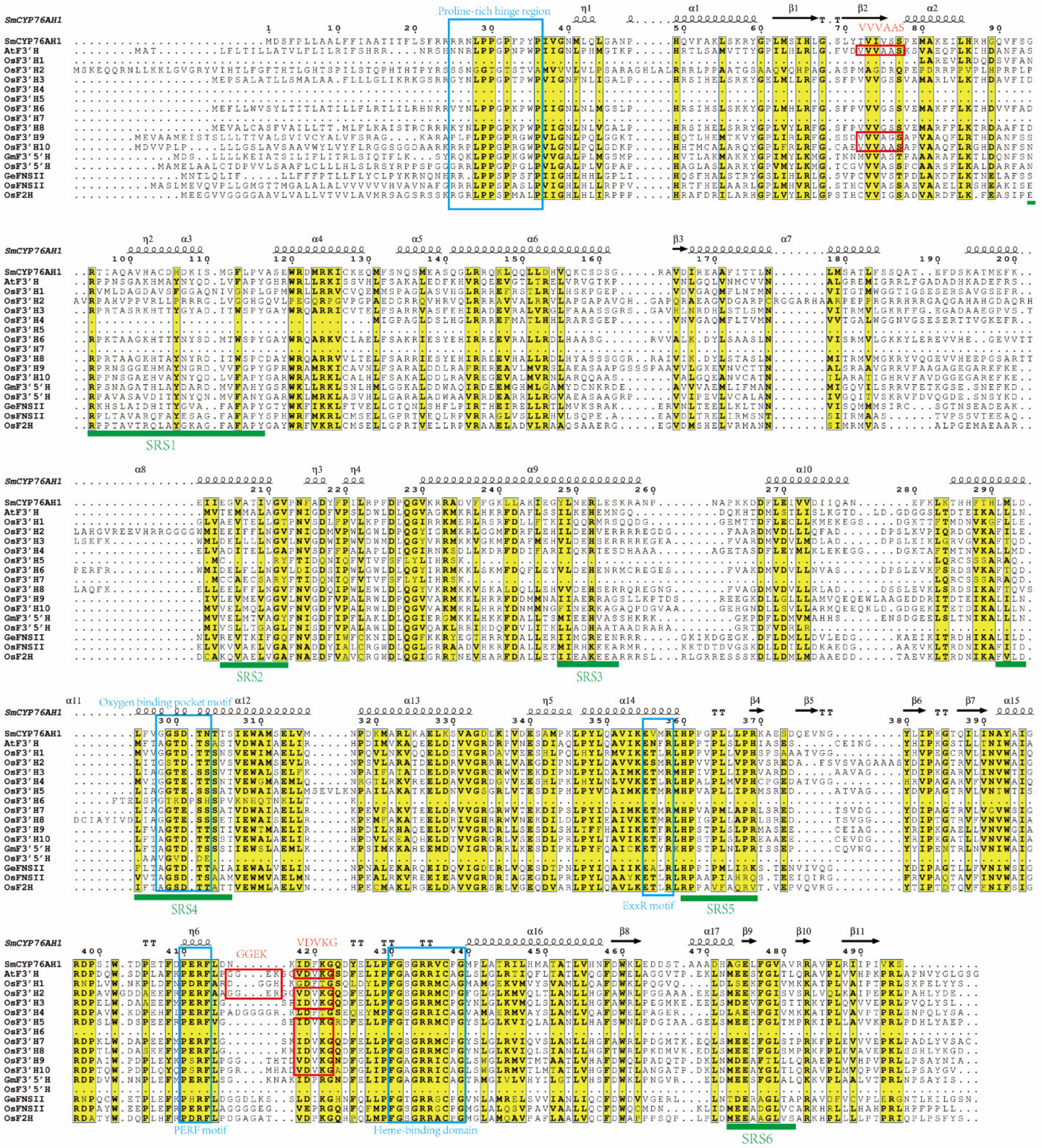

Supplementary Figure S7. Protein sequence alignment of flavonoid biosynthesis-related CYP450s, including F3′Hs, F3′5′Hs, FNSIIs and F2H. The first line indicated the secondary structure of *Salvia miltiorrhiza* CYP76AH1 (SmCYP76AH1). The yellow region represented relatively conserved regions. The CYP450-fetured conserved motif, including the proline-rich hinge region, the oxygen-binding pocket motif, ExxR motif and PERF motif, and the heme-binding domain were boxed in light-blue. Residues relevant for F3′H –specific motifs and Substrate recognizatioin sites (SRSs) were marked with red boxes and green underlines, respectively.

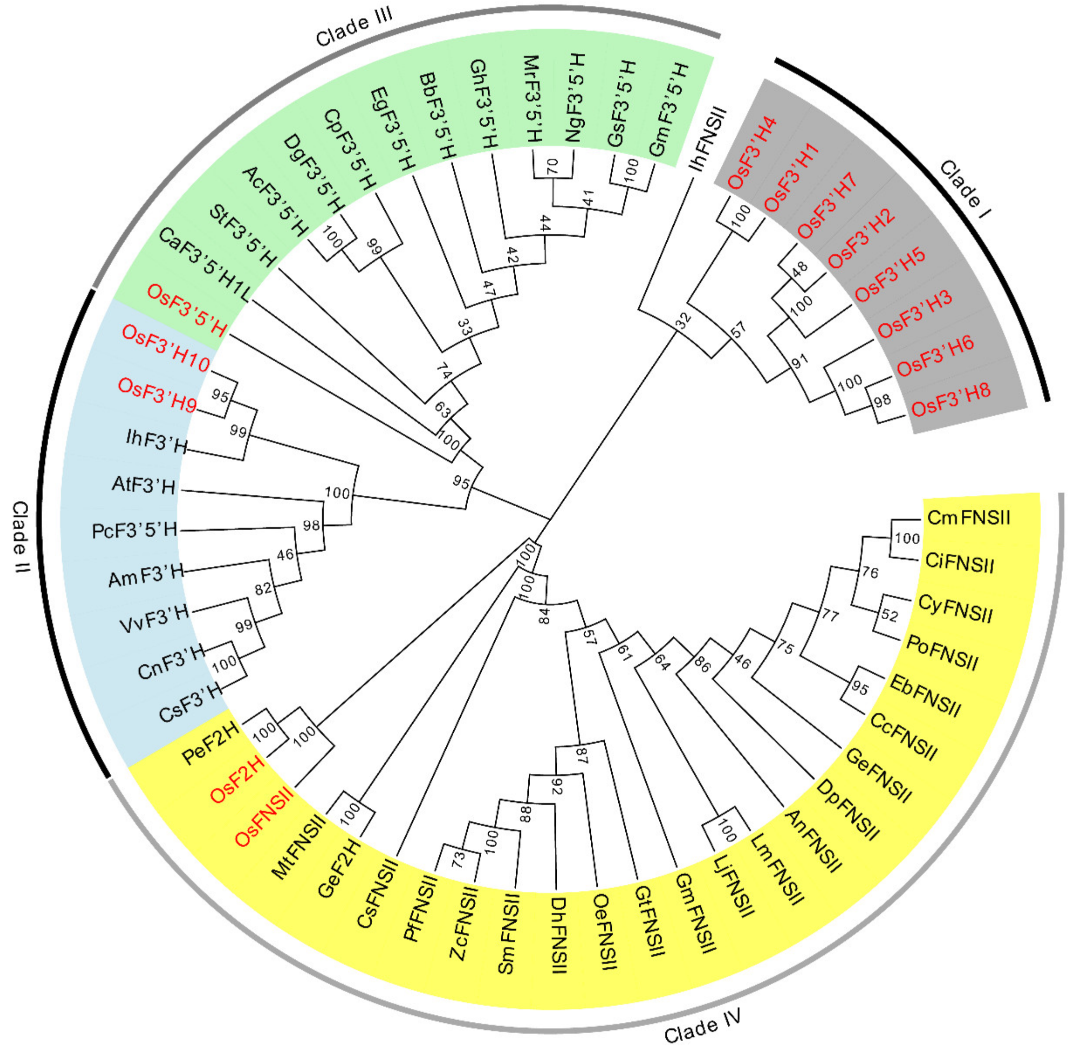

Supplementary Figure S8. Phylogenetic tree of flavonoid biosynthesis-related CYP450s, including F3'Hs, F3'5'Hs, FNSIIs and F2Hs. The unrooted neighbor-joining tree was established by the MEGA7.0 program with 1000 Bootstrap replicates. The numbers at the nodes indicate the bootstrap values. Different colors of arcs represented distinct clades. These F3'Hs, F3'5'Hs, FNSIIs and F2Hs used for phylogenetic tree, were obtained from NCBI and their accession numbers were list in Supplementary Table S4.

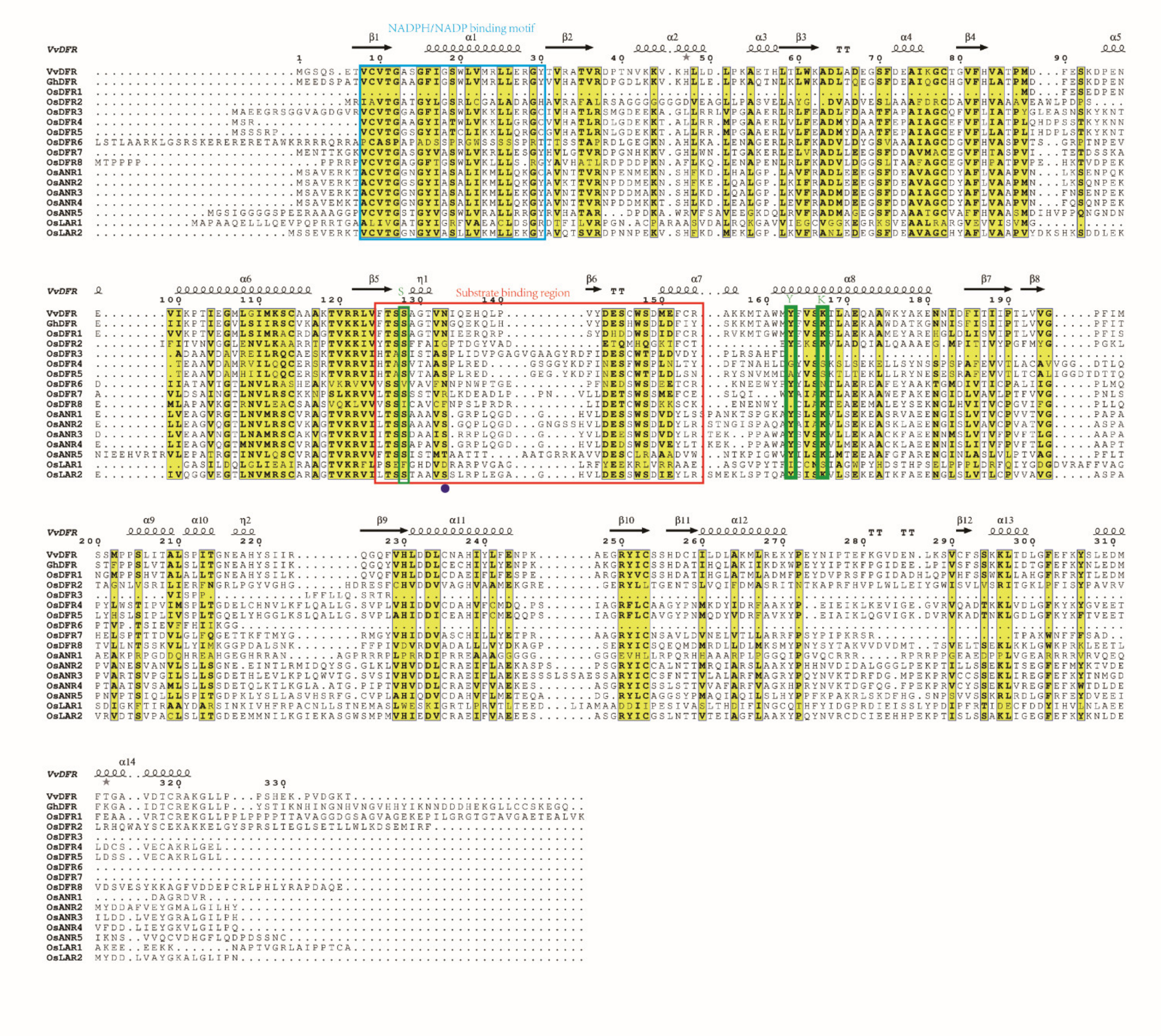

Supplementary Figure S9. Protein sequence alignment of flavonoid biosynthesis-related SDRs, including DFRs, LARs and ANRs. The first line indicated the secondary structure of *Vitis vinifera* DFR (VvDFR). The yellow region represented relatively conserved regions. Residues responsible for glucine-rich Rossmann dinucleotide (NADH/NADPH)-binding motif, the conserved catalytic triad (S128-Y163-K167), and the substrate binding region were boxed with light-blue, green and red respectively. The residues important for the substrate specificity were remarked with dark-blue circles.

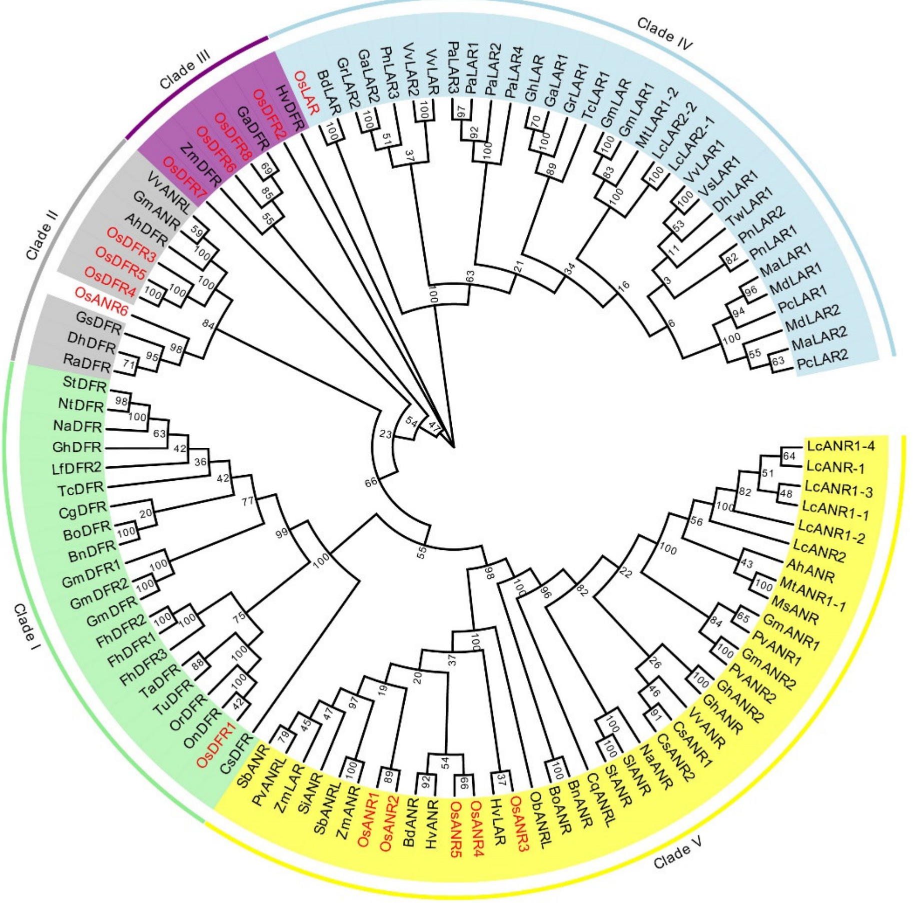

Supplementary Figure S10. Phylogenetic tree of DFRs, LARs and ANRs. The unrooted neighbor-joining tree was established by the MEGA7.0 program with 1000 Bootstrap replicates. The numbers at the nodes indicate the bootstrap values. Different colors of arcs represented distinct clades. These flavonoid biosynthesis-related SDR proteins including DFRs, LARs and ANRs used for phylogenetic tree, were obtained from NCBI and their accession numbers were list in Supplementary Table S5.
